# The use of specific anti-growth factor antibodies to abrogate the oncological consequences of transfusion in head & neck squamous cell carcinoma: an *in vitro* study

**DOI:** 10.1186/1758-3284-4-22

**Published:** 2012-05-16

**Authors:** Tahwinder Upile, Waseem Jerjes, Sandeep Singh, Mohammed Al-Khawalde, Zaid Hamdoon, Hani Radhi, Colin Hopper

**Affiliations:** 1 Department of Head and Neck Surgery, Chase Farm & Barnet NHS Trust, Enfield, UK; 2 Head & Neck Unit, University College London Hospital, London, UK; 3 Department of Surgery, School of Dentistry, Al-Yarmouk University College, Baghdad, Iraq; 4 Oral and Maxillofacial Surgery Unit, AL-Mustansirya University's, Baghdad, Iraq; 5 UCL Department of Surgery, University College London, London, UK; 6 Leeds Institute of Molecular Medicine, Leeds, United Kingdom; 7 Oral and Maxillofacial Surgery Unit, Royal Medical Services, Amman, Jordan; 8 Department of Surgery, School of Dentistry, University of Mosul, Mosul, Iraq; 9 Unit of Oral and Maxillofacial Surgery, UCL Eastman Dental Institute, London, United Kingdom

## Abstract

**Introduction:**

Perioperative blood transfusion is associated with reduced prognosis in a number of solid malignancies. We investigate its role in a head & neck squamous cell cancer (HNSCC) cell lines. Growth of these cell lines was analogous to endothelial growth. Direct exposure to transfusion products exaggerated this effect. It was logical therefore to assess the effects of anti-endothelial antibodies on this interaction.

**Materials and methods:**

Control (HUVEC) and tumour cell lines were exposed to transfusion products. The pre-incubation of the transfusion product with anti-endothelial growth factors was assessed by a growth assay. Where appropriate cells were pre-incubated for 1 hour with 10 μl of a mixture of 100 μl of each and anti-ligand antibodies, the corresponding blood product supplement was incubated with 10 μl of a mixture of 100 μl each of anti-ligand antibodies 1 hour before supplementation to the appropriate cell line. All results are representative of at least two independent experiments carried out in triplicate.

**Results:**

The antibody did not directly reduce growth in the tumour cell line, however there was a significant reduction (p < 0.001) in tumour cell line vascular mimicry caused by transfusion products pre-incubation with anti-endothelial growth factor antibody. This was found in several other tumours.

**Conclusion:**

Perioperative blood transfusion is associated with reduced prognosis in a number of solid malignancies including HNSCC. However this phenomenon is abrogated by the use of anti-endothelial growth factor antibodies. This suggests that the original effect was mediated by the endothelial growth factor family.

## Introduction

The perception of blood transfusion in the perioperative setting has moved from a benign intervention, occasionally life saving, to an outcome to be avoided. The recognition in the mid 1980’s that blood transfusion carried the risk of HIV infection forced a re-evaluation of the indications for transfusing a surgical patient [1]. Perioperative blood transfusion was also found to be associated with reduced prognosis in a number of solid malignancies [2].

Transfusion is in essence a transplant of allogenic cells and its risks are not negligible. Allogeneic blood transfusion is the most frequent allo-transplantation procedure performed on a routine basis with no prior HLA-typing. 50% of the recipients of unprocessed red cells and platelets become allo-immunised [3]. The potential for transmission of unidentified viruses is unknown. The deleterious consequences of blood transfusion arise from many sources. Transfusion is also known to be immunosuppressive, and is an independent risk factor for nosocomial infection and the recurrence of malignancy [4]. Blood is also an increasingly scarce resource.

A link between perioperative blood transfusion and worsened cancer prognosis was first proposed by Francis in the Lancet [5]. Since then it has been established in colorectal, cervical, breast and prostate cancer.

The tumour effects that worsen prognosis are tumour growth and cancer spread. These are now thought to be facilitated in some way by transfusion, causing the overall prognosis to decline [1-5].

Such facilitation may be active (i.e. a direct effect) or passive (i.e. an indirect immuno-depressive effect). Literature and previous research has focused upon the later passive effect, regarding a blood transfusion as simply a means of delivering oxygen carrying capacity and volume expansion with little regard either to the other cells and components also transfused within the packed red blood cells or even the non-haemoglobin contents of the red cells themselves [3-8].

Determining the adverse effects of perioperative transfusion is difficult in head and neck cancer patients because of several confounding factors. Often the mucosal disease process causes cachexia (not only through dysphagia) with its effect on local tropic mucosal and systemic immunity allowing tumour progression. Some preliminary studies have occurred in the head and neck but the results from these were conflicting due mainly to the lack of consideration to confounding factors [6-8]:

A) The patients are severely compromised anyway, and that the group is self selected because only the more prognostically challenged patients are likely to require a transfusion [6–8].

This is supported from studies upon cancer of the colon, rectum, cervix and prostate, Blumberg concluded that transfusion of whole blood may represent a surrogate marker for advanced or more aggressive clinical disease [6-8].

B) The Transfusion has an immune modulating effect (similar to a graft versus host effect) allowing the tumour to escape host immuno-surveillance [6–8].

It was found that allogeneic transfusions were associated with increase in cancer recurrence rates (80% in colorectal cancer) and postoperative bacterial infections (200% to 1000%). A possible mechanism was suggested to be anergy due to presence of large amounts of antigens. This immune dys-regulation caused by transfusion augmented by the effects of haemorrhage, anaesthesia, and surgical stress combine to create an adverse overall effect [9].

Previous efforts to explain the possible effects of blood transfusion on the recurrence of colorectal cancer have been based entirely on the immunosuppressive effects of blood transfusion. However the relationship between solid tumour development and the immune system is inconclusive. Investigators did not take into account that transfusion may have a direct effect on tumours [10].

The common and erroneous assumptions about blood transfusions include the ideas that a unit of red blood cells contain only red cells, that red cells only carry oxygen and carbon dioxide, and that platelets provide only clotting factors. In fact, pure blood products are rarely clinically achievable and most transfusions, even leuco-depleted packed red cells, contain a mixture of components. These include a significant number of white blood cells (monocytes and neutrophils) and platelets, some of which contain high concentrations of physiologically biologically active substances [11]. The different blood product components contain these substances to different degrees [12-16].

The ‘red cell lesion’ of ATP depletion and cell membrane dysfunction with age-related fragility has been known for some time [17], as well as the fact that particularly platelets contain many biologically active substances. It is believed that as result of normal physiological aging and metabolic processes with depletion of ATP and reduction of active membrane processes, there is leaching of biologically active substances from the cells into stored blood [18]. These leached bioactive substances which include epidermal growth factor (EGF), fibroblast growth factor (FGF) and vascular endothelial growth factor (VEGF) have immuno-modulatory effects, which may in part explain the increased likelihood of postoperative sepsis [19-25] and adult respiratory distress syndrome in transfusion recipients [20,22]. They also promote cell growth and angiogenesis, and may therefore have a direct effect on tumour growth [2].

Given the above, it is proposed that the extra-cellular accumulation of bioactive factors in blood transfusion products can directly [2] and indirectly cause tumour growth and hence a worsening of prognosis.

Cancer Data from patients with colon, rectum, cervix and prostate cancer showed an association between transfusion, of any amount of whole blood, or larger amounts of packed red blood cells, and later recurrence of cancer. Recipients of any amounts of whole blood had similar recurrence rates (38-52%). Proportional hazard risk analysis showed that transfusion of any whole blood or more than three units of red blood cells was significantly associated with earlier recurrence and death due to cancer [26].

We investigate the role of blood product transfusion in a head & neck squamous cell cancer (HNSCC) cell line. Growth of these cell lines was analogous to endothelial cell line growth. Direct exposure to transfusion products exaggerated this effect. It was logical therefore to assess the effects of anti-endothelial antibodies on this interaction.

## Materials and methods

Control (HUVEC) and tumour cell lines were exposed to transfusion products. The pre-incubation of the transfusion product with anti-endothelial growth factors was assessed by a growth assay [2].

The cell lines used were HUVEC, HN5a, 5b, 11a, 11b, HCT116, DU145. Cells were grown in culture in culture flasks (Costar 75 cm^2^ Vent Cap) in their appropriate growth media (DMEM with 10% Fetal Calf Serum, 5% CO2). Cells were grown to 75% confluence and serum starved for 24 to 48 hours before use. Experiments were carried out using the appropriate serum free medium for each cell line. Mycoplasma assays were carried out to exclude infection [27,28].

Pilot experiments were undertaken to assess growth of a bank of tumour cell lines on (growth factor reduced) matrigel (Sigma) with standard media (DMEM with 10% Fetal Calf Serum).

The cells were incubated at 37°C with 5% CO2. Phase contrast photomicrographs (Olympus systems) were taken at 4 hours and 24 hours. Image analysis was performed by using Image Pro Plus 4.0 computer image analysis software, particular features of interest were two dimensional area (surrogate of growth and migration), branch points and end point measurements (surrogate of intercellular complexity).

A modified growth assay was undertaken cells whereby cells were grown to 75% confluence and plated directly upon a 96 well plate at a seeding density of 2*103 cells per well, and serum starved for 24 hours. The cells were then grown in serial dilutions of either pure antibody mixture or blood product or a combination for a further 48 hours. 100 μl of alkaline phosphates substrate (3 mg/ml) was then added to each well, incubated for 2 hours after which 50 μl of 1 M NaOH was added and the plate placed in a microplate reader and read at 405 nm wavelength.

Where appropriate cells were pre-incubated for 1 hour with 10 μl of a mixture of 100 μl of each and anti-ligand antibodies, the corresponding blood product supplement was incubated with 10 μl of a mixture of 100 μl each of anti-ligand antibodies 1 hour before supplementation to the appropriate cell line. All results are representative of at least two independent experiments carried out in triplicate.

## Results

**Figure 1:**
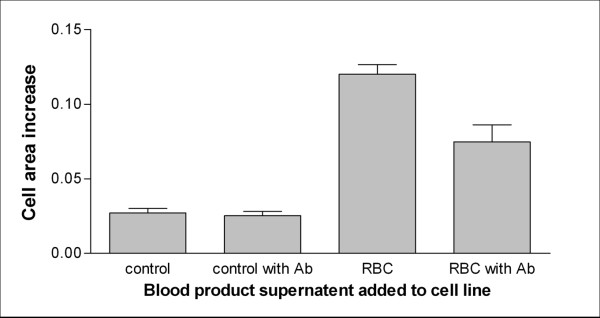
**Graph showing area increase (mm**^**2**^**) of an endothelial cell line (growth by vascular mimicry) after supplementation with stored blood transfusion packed red cell products (pre-incubated with anti-endothelial growth factor antibodies).** There was a significant difference between growth of control samples and cell lines supplemented with supernatant from blood transfusion products (p < 0.001) as well as a significant difference between the effect of pre-incubation of supernatant with anti-endothelial growth factor antibodies on cell line growth (p < 0.001) with control and pure supernatant samples.

**Figure 2:**
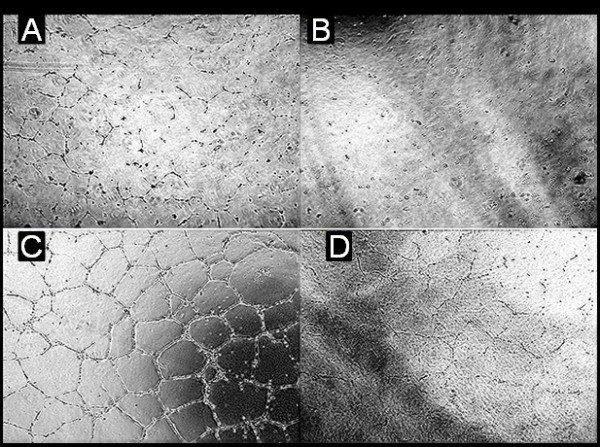
**Showing the effect of the addition of blood product supernatant upon the growth of an endothelial cell line (HUVEC).A** (top left) represents a control group; **B** (top right) represents the control group with the addition of anti-endothelial growth factor antibody; **C** (bottom left) represents the effect of incubation with blood product supernatant; **D** (bottom left) represents the effect on HUVEC growth in media supplemented with blood product supernatant pre-incubated with anti endothelial growth factor. This is representative of the direct effects of blood product supernatant upon the cell lines studied. The effect of anti-endothelial growth factor antibodies is similar, i.e. in reducing vascular channel formation. There was a significant statistical difference between groups represented by ‘A, C and D’, but not between groups ‘A and B’ suggesting there is a direct effect of supplementation with blood product supernatant which is abrogated by the use of anti-endothelial growth factor antibody but no cytotoxic effect of the antibody per-se on the cell lines.

The supernatant of stored blood transfusion products causes increased growth in the cell lines investigated, both control vascular and tumour cell line (ﻼ^2^, p < 0.001). The antibody did not directly reduce growth in the cell line, however there was a significant reduction((ﻼ^2^, p < 0.001) in cell line growth caused by transfusion products pre-incubation with anti-endothelial growth factor antibody. This was also found in the other tumour cell lines investigated, (Figures 1 and 2). This suggests that there is a direct “growth and vascular mimicry promotion” effect of supplementation with blood product supernatant which is abrogated by the use of anti-endothelial growth factor antibody but no cytotoxic effect of the antibody per-se on the cell lines. This was confirmed by separate growth assay.

## Discussion

**Figure 3:**
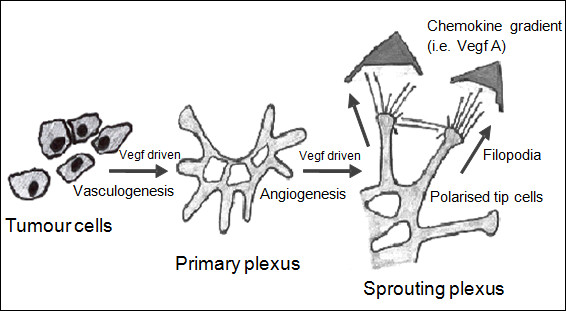
Showing a diagrammatic representation of the direct effect of growth factors in the supernatant of blood products, which promote growth, vasculogenesis and angiogenesis.

Our results, suggest a novel hypothesis, that there is a direct “growth and vascular mimicry promotion” effect of the supplementation of HNSCC cell lines with blood product supernatant (Figure 3). This is specifically abrogated by the use of anti-endothelial growth factor antibodies.

Furthermore since HNSCC is known to secrete endothelial growth factors (both in the serum and saliva) which may act in an autocrine manner of residual tumour cells [27,28], it seems a logical extension to hypothesize that there is a direct growth effect caused by exposure to similar growth factors leached from the anucleate red cells, contaminating white cells and platelets which are all found in a “unit” of “leukocyte” depleted packed red cells.

We have shown some of the prognostically deleterious effects of perioperative transfusion in head & neck cancer patients are caused by the transfusion product’s release of endothelial growth factors. This was also found to be the case in several of the tumour groups (colonic and prostate) for which this phenomenon has been previously reported. It can now be hypothesized that this is due to the specific expression of receptors to these growth factors in these tumour types which are not universally found. This would explain why this phenomenon does not occur for all tumour types.

## Conclusion

We wound tentatively suggest the following clinical implications and adjustments to practice. If possible we would avoid transfusions by meticulous technique and haemostasis, plasma expansion and oxygenation. Alternatives to the antiquitidated "10/30" transfusion trigger (i.e. haemoglobin less than 10 g/dL or haematocrit less than 30) should be sought including the use of synthetic blood analogues and recombinant erythropoietin. If transfusion is inevitable then the minimal necessary amount, to alleviate symptoms with due consideration to the patients pre-morbid cardio-respiratory status, should be given. The use of specific anti-endothelial growth factor antibody filters before transfusion may be mooted although simple red cell washing before transfusion may be simpler and more cost effective.

## Competing interests

The authors declare that they have no competing interests.

## Authors’ contributions

TU,WJ, SS, MA, ZA,HR and CH designed the study, carried out the literature research and manuscript preparation. TU,WJ, SS, MA, ZA,HR and CH were responsible for critical revision of scientific content and manuscript preparation and review. All authors contributed to conception and design and approved the final version of the manuscript.
